# Adherence to the Mediterranean diet and colorectal cancer risk: a large case control study in the Moroccan population

**DOI:** 10.1017/S1368980025000199

**Published:** 2025-02-06

**Authors:** Khaoula El Kinany, Zineb Hatime, Achraf El Asri, Abdelilah Benslimane, Meimouna Mint Sidi Deoula, Btissame Zarrouq, Pagona Lagiou, Karima El Rhazi

**Affiliations:** 1 Higher Institute of Nursing Professions and Health Techniques, Fez, Morocco; 2 Department of Epidemiology and Public Health, Faculty of Medicine and pharmacy of Fez, Sidi Mohamed Ben Abdellah University, Fez, Morocco; 3 Department of Biology and Geology, Teachers Training College (Ecole Normale Superieure), Sidi Mohamed Ben Abdellah University, Fez, Morocco; 4 Department of Hygiene, Epidemiology and Medical Statistics, School of Medicine, National and Kapodistrian University of Athens, Athens, Greece; 5 Department of Epidemiology, Harvard T.H. Chan School of Public Health, Boston, MA, USA

**Keywords:** Mediterranean diet, Mediterranean diet score, Colorectal cancer, Case–control, Morocco

## Abstract

**Objective:**

The Mediterranean diet (MD) is a dietary pattern associated with several health benefits, including reduction of risk for various cancers. We conducted a study to investigate associations between adherence to the MD and colorectal cancer (CRC) subtype risk among Moroccan adults.

**Design:**

A matched case–control study.

**Setting:**

The five major university hospitals in Morocco.

**Participants:**

A total of 3032 subjects (1516 CRC patients and 1516 controls) matched on age, sex and centre were recruited between September 2009 and February 2017 at five major hospitals in Morocco. Diet was assessed using a validated FFQ. Adherence to the MD was assessed through a score, ranging from 0 (no adherence) to 10 (maximal adherence) and divided into three categories (low, middle and high). Conditional logistic regression was performed to calculate multivariable OR and 95 % CI with low adherence (score 0–3) as referent, adjusting for potential confounding factors.

**Results:**

Close adherence to the MD (score 6–9) was associated with reduced risk of CRC (ORa = 0.74, 95 % CI 0.63, 0.86), rectal cancer (ORa = 0.73, 95 % CI 0.58, 0.90) and colon cancer (ORa = 0.74, 95 % CI 0.60, 0.92).

**Conclusion:**

Our study, conducted in a southern Mediterranean population, adds to the evidence suggesting a protective effect of MD against CRC risk.

Colorectal cancer (CRC) is the third common cancer diagnosed worldwide with nearly 1.93 million new cases and 904 019 deaths estimated in 2022^(1)^. There is a large geographic difference in the global distribution of CRC. The highest incidence is observed in Europe^(2)^, whereas the lowest incidence in the North Africa^(3)^. In Morocco, CRC incidence was 2752 and 2554 in men and women, respectively, in 2022^(4)^. The development of CRC is a multi-factorial disease process, with contributions from diet, physical activity and body weight, according to the latest report of the World Cancer Research Fund and the American Institute for Cancer Research^(5)^.

To explore the association between diet and chronic diseases, different scores for the assessment of adherence to dietary patterns have been developed and updated^(6–8)^. The traditional Mediterranean diet (MD) has been associated with favourable health effects, including longevity and non-communicable disease risk reduction^(9–13)^. According to the most updated version of the MD pyramid^(14)^, numerous components like cereals, vegetables, legumes, fruits and nuts, fish and olive oil have been identified as beneficial for overall health. Moreover, epidemiological studies repeatedly demonstrate a potential protective effect of MD against CRC risk^(15,16)^.

During the last decades, a transition in the dietary habits worldwide, including Mediterranean countries like Morocco, has been illustrated in several epidemiological studies^(17–19)^. In the Mediterranean region, there has been a noticeable transition characterised by a move away from traditional Mediterranean dietary practices towards adopting a more Westernized approach to eating habits^(20)^.

To our knowledge, there has only been one study that investigated the association between adherence to a modified MD score and overweight/obesity risk in Moroccan adults^(21)^. As for the association of MD with CRC risk in the Moroccan population, this has not yet been studied. This study is the first large case–control one conducted in Morocco to assess the association between adherence of the Moroccan population to the MD and CRC subtypes risk.

## Materials and methods

The details of the study methodology have been previously published^(22)^. In summary, this study was a gender, age and centre-matched case–control conducted between September 2009 and December 2017 in five major Moroccan University Hospital centres located in Rabat, Casablanca, Marrakech, Oujda and Fez.

Cases were patients with a recent, anatomo-pathologically confirmed the diagnosis of CRC. Controls were healthy subjects from the same population from which the cases arose.

Recruitment began after the clear explanation of the study objectives. Informed consent was obtained from all participants, and the confidentiality of their data was maintained. In brief, data collection included socio-demographic characteristics (age, sex, residency, monthly income and marital status), anthropometric measurements (weight, height and BMI), physical activity intensity (low, moderate and high), smoking status (never smokers, ex-smokers and current smokers), alcohol consumption (yes or no), family history of CRC (yes or no) and past regular consumption of non-steroidal anti-inflammatory drug (yes or no).

### Dietary assessment

Dietary information over the previous 12 months was collected using a validated FFQ that contained 255 items to ensure the representability of local dietary habits^(23)^.

### Mediterranean diet score

To assess adherence to the traditional MD, an MD score was constructed on the basis of the MD score established by Trichopoulou et al^(24)^. Ten components were included in this MD score: Cereals (wheat, oat, barley, seigle, spelt, semolina, corn and rice), dairy products (milk, lben, yaourt, ice cream and cream), legumes (all types of beans, lentils and all types of peas), fruits and nuts (all fruits, olives, dates and all types of nuts), vegetables (all vegetables), fish (any fish fresh and seafood), poultry (chicken and turkey), red meat (red meat and processed meat), alcohol (any type of beer, of wine and other alcoholic beverages like port, sherry, liquor and spirits) and MUFA/ SFA ratio: this ratio presents the fatty intake. This ratio was estimated as follows: for MUFA estimation, we used olive oil consumption, which is the main component of MUFA and it is the main source of MUFA in Mediterranean populations^(25,26)^. It is also consumed in high quantities in Moroccan population because of its relatively low price. For SFA, we calculated the sum of fatty acid proportions included in each food category of the FFQ. The food composition table available in Morocco was used to derive the nutrient composition of several traditional dishes and some modern products^(27)^. Other regional data were used to extract the composition, namely the Tunisian food composition table^(28,29)^, the food composition table for African countries (FAO) and the French food composition table.

The sex-specific median consumption was calculated for each component in controls as reference population. For components not frequently consumed in the context of the MD (red meat, poultry, dairy products and alcohol), we attributed, for each participant, a value of 0 if her/his consumption was equal to or above the median and a value of 1 if it was under the median. For components frequently consumed in the context of the MD (cereals, legumes, fruits and nuts, vegetables, MUFA/SFA ratio and fish), a value of 1 was attributed for consumption equal to or above the median and a value of 0 for consumption under the median. The MD score for each subject was the sum of the ten component values. Thus, the total MD score ranged from 0 (minimal adherence to the MD) to 10 (maximal adherence).

### Statistical analysis

Comparison between cases and controls were performed for all variables using the Mac Nemar χ^2^ test. MD score was categorised as low (0–3 points), medium (4–5 points) and high (6–10 points), with the high score denoting closer adherence to the MD. CRC cases and controls were individually matched on age (±5 years), sex and centre. Conditional logistic regression models were performed to compute OR and 95 % CI and to estimate the association between different categories of MD score (low, middle and high) and the CRC risk overall and by anatomic location (colon, rectum separately and CRC overall) for all population and using sex-specific cut-off for separated analysis. Potential confounding factors (age (in years because matching has not removed age confounding), area of residence (urban and rural), education level (illiterate, primary, secondary and higher), monthly income (low, medium and high), smoking status (never smoker, ex-smoker and current smoker), BMI categories (normal, underweight, overweight and obesity), physical activity (high intensity (≥3000 MET-minutes per week), moderate intensity (600–3000 MET-minutes per week) and low intensity (<600 MET-minutes/week)), and total energy intake (continuous, kcal/day)) was included in the adjusted model. Data analyses were performed using SPSS 20.0 version. A *P* value of 0.05 or lower was considered statistically significant.

## Results

Table 1 presents general characteristics. The mean age at recruitment was 56.4 ± 13.9 years for cases and 55.5 ± 13.7 years for controls. The majority of the study population lived in urban area with (69.2 % *v*. 75.7 %; *P* < 0.05) for cases and controls, respectively. Controls had a significantly better educational level compared with cases. Most of the participants presented a modest monthly household income. The smoking status differed significantly between cases and controls, respectively, with (77.6 % *v*. 83.8 %; *P* < 0.05) for never smokers, (12.10 % *v*. 6.20 %; *P* < 0.05) for smokers. Concerning physical activity, controls were significantly more active than cases with (26.6 % *v*. 21.7 %; *P* < 0.05) for the higher level of physical activity, (22.2 % *v*. 33.9 %; *P* < 0.05) for the lower one, respectively. Finally, cases were clearly more obese than controls with (15.8 % in cases *v*. 8.7 % in controls, *P* < 0.05).

**Table 1.** General characteristics of the study population (*n* 2906)

		Cases	Controls
Data		*n*(%)	*n*(%)
Centre	Rabat	479 (33.00)	479 (33.00)
	Casablanca	475 (32.70)	475 (32.70)
	Oujda	249 (17.13)	249 (17.13)
	Fez	224 (15.40)	224 (15.40)
	Marrakech	26 (01.77)	26 (01.77)
Sex	Female	737 (50.70)	737 (50.70)
	Male	716 (49.30)	716 (49.30)
Marital status	Single	136 (9.40)	141 (9.7)
	Married	1109 (76.3)	1119 (77.0)
	Divorced	46 (3.2)	56 (3.9)
	Widow	162 (11.1)	137 (9.4)
Area of residence ^*^	Urban	1005 (69.2)	1100 (75.7)
	Rural	448 (30.8)	353 (24.3)
Educational level^*^	Illiterate	918 (63.2)	729 (50.2)
	Primary	274 (18.9)	272 (18.7)
	Secondary	175 (12.0)	267 (18.4)
	Higher	86 (5.9)	185 (12.7)
Monthly household income (MAD)^*^	<2000	1200 (82.6)	1044 (71.9)
	2000–5000	204 (14.0)	289 (19.9)
	>5000	49 (3.4)	120 (8.3)
Smoking status^*^	Never smokers	1127 (77.6)	1217 (83.8)
	Ex-smokers	150 (10.3)	146 (10.0)
	Smokers	176 (12.1)	90 (6.2)
Family history of colorectal cancer^*^	No	1373 (94.5)	1441 (99.2)
	Yes	80 (5.5)	12 (0.8)
Alcohol consumption	No	1418 (97.6)	1428 (98.3)
	Yes	35 (2.4)	25 (1.7)
		Mean ± sd	Mean ± sd
Age (years)^*^		56.45 ± 13.95	55.50 ± 13.70
Energy intake (kcal)^*^		3240.14 ± 826.14	3245.10 ± 833.75

* *P* < 0.05; MAD: Moroccan Dirham.

Table 2 presents the sex-specific median of MD component intake among controls. Compared with women, men consumed more legumes, fruits and nuts, fish and also more red meat and dairy products. Medians observed for cereals, vegetables, poultry and alcohol intake did not differ between men and women.

**Table 2.** The sex-specific median of Mediterranean diet component intake among controls

Median of controls		
Components	Females	Males
Cereals (g/day)	449.40	453.15
Vegetables (g/day)	240.07	238.26
Legumes (g/day)	126.66	138.33
Fruit and nuts (g/day)	131.70	137.02
Fish (g/week)	149.31	182.00
Ratio MUFA/SFA	1.29	1.29
Red meat (g/week)	215.60	234.22
Poultry (g/week)	308.00	308.00
Dairy products (g/day)	142.66	157.80
Alcohol (g/day)	0.00	0.00
Energy intake (kcal/day)	3247.19	3394.77

Table 3 presents the distribution of cases and controls by MD score category, overall and by gender. Controls appear to adhere more closely to MD than cases (57.6 % *v*. 42.4 %; *P* < 10^−3^, respectively, for the high score category among both genders).

**Table 3.** Distribution of cases and controls by Mediterranean diet (MD) score category: results overall and by gender

		Cases n (%)	Controls n (%)	*P*	Cases n (%)		Controls n (%)		*P*
		Total population			Men	Women	Men	Women	
MD SCORE	Low 0–3 points	325 (54.4)	272 (45.6)	10^−3^	158 (54.9)	167 (54.0)	130 (45.1)	142 (46.0)	10^−3^
	Middle 4–5 points	700 (53.8)	600 (46.2)		339 (53.4)	361 (54.3)	296 (46.6)	304 (45.7)	
	High 6–10 points	428 (42.4)	581 (57.6)		219 (43.0)	209 (41.8)	290 (57.0)	291 (58.2)	

Table 4 shows conditional logistic regression derived OR and 95 % CI for CRC, overall and by subsites, per category of MD score. Inverse associations were found between the adherence to the MD and CRC risk. Comparing the high *v*. the low score categories, there was a 26 % reduction of CRC (ORa = 0.74, 95 % CI 0.63, 0.86). The results for colon and rectal cancer were ORa = 0.74, 95 % CI 0.60, 0.92 and ORa = 0.73, 95 % CI 0.58, 0.90, respectively. For CRC overall, results were similar in the separate analysis for men (ORa = 0.74, 95 % CI 0.59, 0.92) and women (ORa = 0.73, 95 % CI 0.59, 0.91). For colon cancer, the inverse associations were statistically significant and more pronounced among men (ORa = 0.67, 95 % CI 0.49, 0.92) than women (ORa = 0.80, 95 % CI 0.59, 1.07), whereas the opposite was true for rectal cancer (for women ORa = 0.65, 95 % CI 0.48, 0.90; for men ORa = 0.78, 95 % CI 0.58, 1.05).

**Table 4.** OR and CI for colorectal cancer (CRC) overall and by subsites per category of Mediterranean diet (MD) score

MD SCORE	CRC Overall (*n* 1453)				Colon Cancer (*n* 729)				Rectal Cancer (*n* 724)			
	OR_c_	95 % CI	OR_a_	95 % CI	OR_c_	95 % CI	OR_a_	95 % CI	OR_c_	95 % CI	OR_a_	95 % CI
Total population												
Low (0–3 points)	1		1		1		1		1		1	
Middle (4–5 points)	0.98	0.86, 1.12	0.96	0.84, 1.09	0.96	0.80, 1.15	0.93	0.77, 1.12	1.02	0.84, 1.23	0.97	0.80, 1.17
High (6–10 points)	0.77	0.67, 0.90	0.74	0.63, 0.86	0.78	0.64, 0.95	0.74	0.60, 0.92	0.77	0.63, 0.95	0.73	0.58, 0.90
Women												
Low (0–3 points)	1		1		1		1		1		1	
Middle (4–5 points)	1.00	0.83, 1.20	0.97	0.81, 1.17	1.01	0.79, 1.30	0.99	0.77, 1.28	0.98	0.75, 1.29	0.93	0.70, 1.22
High (6–10 points)	0.77	0.63, 0.94	0.73	0.59, 0.91	0.82	0.62, 1.09	0.80	0.59, 1.07	0.71	0.53, 0.96	0.65	0.48, 0.90
Men												
Low (0–3 points)	1		1		1		1		1		1	
Middle (4–5 points)	0.97	0.80, 1.17	0.94	0.78, 1.14	1.01	0.79, 1.30	0.85	0.65, 1.12	1.05	0.80, 1.37	0.99	0.76, 1.30
High (6–10 points)	0.78	0.63, 0.96	0.74	0.59, 0.92	0.82	0.62, 1.09	0.67	0.49, 0.92	0.83	0.62, 1.11	0.78	0.58, 1.05

Crude odds ratio (ORc); crude model adjusted for age and total energy intake.

Adjusted odds ratio (ORa): Assessed by analysing CRC cases and their individually matched controls by conditional logistic regression, conditioning for matching factors (age, sex and centre) and adjusted for age, area of residence, educational level, monthly income, family history of CRC, smoking status, BMI, physical activity and energy intake.

## Discussion

Our results indicate that closer adherence to the traditional MD is associated with reduced CRC risk, overall and by colon and rectal subtypes. In the analyses by sex, the inverse association was similar for men and women for CRC overall, whereas regarding subtypes the inverse associations were more pronounced and statistically significant among men for colon cancer and among women for rectal cancer.

Several studies have reported a potentially protective effect of MD on CRC risk^(6,30,31)^. This effect has been attributed to the main components of MD, such as olive oil, rich in MUFA and squalene, cereals, fruits and vegetables rich of phytosterols, phenols and dietary fibre that have a significant impact on the prevention of CRC^(5)^, and it contributes to the improvement of the gut microbiota^(32)^.

The pathophysiology of CRC involves several risk factors in men and women through many different pathways. Reports indicate that insulin resistance can cause higher insulin levels with higher body fatness that subsequently impact the colon stability through promotion of inflammatory pathway, cell growth and inhibiting apoptosis^(33)^. Interestingly, as mentioned above, we found that close adherence to the MD protected significantly against colon cancer in men, but the results were NS in women. Similar results were reported in the Italian EPIC cohort^(34)^, showing that the MD protective effect disappeared when only women were considered and persisted for the rectal cancer in subsite analyses. Furthermore, in a recent study, researchers argued that differences in colon cancer aetiology between men and women were related to dietary and behavioural factors, such as high red meat consumption and smoking^(35)^. In our population study, we found that women were significantly more obese than men. According to the Moroccan national survey, women have a higher prevalence of overweight than men, reaching nearly 48 %, and higher visceral fat has been associated with risk of colon cancer development^(36)^. Though in our analyses, we control for BMI, waist-to-hip ratio is not accounted for, and visceral fat could be a reason why we do not see the reduction in colon cancer risk notes among men also among women. Another important difference between men and women regarding colon cancer development is that genetic factors associated with colon cancer risk, such as hypermethylation, microsatellite instability, BRAF V600E mutation and CpG island methylator phenotype tumors, comprising 20 % of CRC have more frequently been reported among women^(37)^. Women also have higher frequency of KRAS mutations in codon 12 than men,^(38)^ and this mutation is related to more aggressive cancers^(39)^ and could thus attenuate a possible protective effect of the MD^(40)^.

As also mentioned above, we found the inverse association between the MD and rectal cancer risk was not as pronounced among men, as among women. A multi-centre European cohort study reported no significant association between MD and rectal cancer in both men and women and for all MD scales^(41)^. A large cohort of British women exploring diet and lifestyle in relation to CRC outcomes demonstrated an inverse association between the incidence of rectal cancer and the MD with a little association observed for colon cancer outcomes^(42)^. This study was consistent with our results.

Our study shares the limitations inherent in all case–control studies, which are susceptible to recall and other biases and for in residual confounding cannot be confidently excluded. On the other hand, our study was the first study of its kind in Morocco and North Africa with a relatively large and incident diagnoses of cancer where histopathologically confirmed. Dietary habits were assessed by trained interviewers through the use of a validated FFQ containing items and food groups typically consumed by Moroccan population^(43)^.

## Conclusion

Our study, conducted in a southern Mediterranean population, adds to the evidence suggesting a protective effect of MD against CRC risk. Our findings highlight the need to establish dietary guidelines and public health interventions promoting adherence to the traditional MD in Morocco.

## Supporting information

El Kinany et al. supplementary material El Kinany et al. supplementary material

## Supplementary material

For supplementary material accompanying this paper visit https://doi.org/10.1017/S1368980025000199
