# Supplementary material for: Adherence to the Mediterranean diet and colorectal cancer risk: a large case control study in the Moroccan population
Source: Public Health Nutr. 2025 Feb 6;28(1):e48. doi: 10.1017/S1368980025000199 (PMC11983996; doi:10.1017/S1368980025000199)
Supplement: El Kinany et al. supplementary material [file S1368980025000199sup001.docx]

Table. Distribution of cases and controls by groups of Mediterranean Diet (MD) score

| Food groups | | Colon Cancer (n=729) | | Rectal Cancer (n=724) | | CRC Overall (N=1453) | |
| --- | --- | --- | --- | --- | --- | --- | --- |
|  |  | OR_c_ (95 % CI) | OR_a_ (95 % CI) | OR_c_ (95 % CI) | OR_a_ (95 % CI) | OR_c_ (95 % CI) | OR_a_ (95 % CI) |
| Cereals | <411.1 | 1 | 1 | 1 | 1 | 1 | 1 |
|  | ≥411.1 | 1.15 (0.93-1.41) | 0.73 (0.58-0.91) | 0.99 (0.80-1.21) | 0.85 (0.68-1.06) | 1.06 (0.92-1.23) | 0.73 (0.61-0.88) |
| Dairy products | <236.7 | 1 | 1 | 1 | 1 | 1 | 1 |
|  | ≥236.7 | 0.65 (0.52-0.79) | 0.74 (0.59-0.93) | 0.62 (0.50-0.76) | 0.78 (0.63-0.98) | 0.63 (0.54-0.73) | 0.71 (0.59-0.84) |
| Legumes | <126.7 | 1 | 1 | 1 | 1 | 1 | 1 |
|  | ≥126.7 | 0.87 (0.71-1.06) | 0.85 (0.68-1.05) | 0.85 (0.69-1.05) | 0.91 (0.73-1.13) | 0.86 (0.74-0.99) | 0.88 (0.74-1.05) |
| Fruits and nuts | <106.2 | 1 | 1 | 1 | 1 | 1 | 1 |
|  | ≥106.2 | 0.52 (0.42-0.64) | 0.61 (0.48-0.77) | 0.47 (0.38-0.58) | 0.63 (0.50-0.80) | 0.49 (0.42-0.57) | 0.67 (0.55-0.80) |
| Vegetables | <238.6 | 1 | 1 | 1 | 1 | 1 | 1 |
|  | ≥238.6 | 0.61 (0.49-0.75) | 0.69 (0.55-0.87) | 0.55 (0.45-0.69) | 0.67 (0.53-0.85) | 0.58 (0.50-0.67) | 0.61 (0.51-0.73) |
| Fish | <149.4 | 1 | 1 | 1 | 1 | 1 | 1 |
|  | ≥149.4 | 0.69 (0.56-0.84) | 0.72 (0.58-0.91) | 0.64 (0.52-0.79) | 0.74 (0.60-0.93) | 0.66 (0.57-0.77) | 0.73 (0.61-0.87) |
| Poultry | <44.0 | 1 | 1 | 1 | 1 | 1 | 1 |
|  | ≥44.0 | 1.41 (1.14-1.74) | 1.23 (0.98-1.54) | 1.48 (1.20-1.82) | 1.34 (1.07-1.67) | 1.44 (1.24-1.66) | 1.41 (1.19-1.69) |
| Red meat | <38.1 | 1 | 1 | 1 | 1 | 1 | 1 |
|  | ≥38.1 | 1.78 (1.44-2.20) | 1.89 (1.51-2.38) | 1.39 (1.13-1.71) | 1.40 (1.12-1.74) | 1.57 (1.35-1.82) | 1.70 (1.43-2.03) |
| Alcohol | <0.0 | 1 | 1 | 1 | 1 | 1 | 1 |
|  | ≥0.0 | 3.01 (0.55-5.85) | 3.11 (0.67-6.17) | 2.03 (0.91-4.10) | 1.85 (0.90-3.83) | 1.34 (0.78-2.29) | 1.36 (0.78-2.36) |
| MUFA/SFA | <1.3 | 1 | 1 | 1 | 1 | 1 | 1 |
|  | ≥1.3 | 0.97 (0.79-1.19) | 1.13 (0.91-1.42) | 0.70 (0.57-0.86) | 0.85 (0.67-1.06) | 0.82 (0.71-0.95) | 0.94 (0.79-1.12) |

Crude Odds Ratio (ORc); Crude model adjusted for age and total energy intake.

Adjusted Odds Ratio (ORa): Assessed by analyzing CRC cases and their individually-matched controls by conditional logistic regression, conditioning for matching factors (age, sex and center) and adjusted for age, area of residence, educational level, monthly income, family history of CRC, smoking status, BMI, physical activity, energy intake
